# Anti-inflammatory effects of spermidine in lipopolysaccharide-stimulated BV2 microglial cells

**DOI:** 10.1186/1423-0127-19-31

**Published:** 2012-03-20

**Authors:** Yung Hyun Choi, Hye Young Park

**Affiliations:** 1 Department of Biochemistry, Dongeui University College of Oriental Medicine, Busan 614-714, Republic of Korea; 2 Department of Biomaterial Control (BK21 Program), Graduate School, Blue-Bio Industry RIC and Anti-aging Research Center, Dongeui University, Busan 614-714, Republic of Korea

**Keywords:** Spermidine, Inflammation, NF-κB, Akt, MAPKs

## Abstract

**Background:**

Spermidine, a naturally occurring polyamine, displays a wide variety of internal biological activities including cell growth and proliferation. However, the molecular mechanisms responsible for its anti-inflammatory activity have not yet been elucidated.

**Methods:**

The anti-inflammatory properties of spermidine were studied using lipopolysaccharide (LPS)-stimulated murine BV2 microglia model. As inflammatory parameters, the production of nitric oxide (NO), prostaglandin E_2 _(PGE_2_), interleukin (IL)-6 and tumor necrosis factor (TNF)-α were evaluated. We also examined the spermidine's effect on the activity of nuclear factor-kappaB (NF-κB), and the phosphoinositide 3-kinase (PI3K)/Akt and mitogen-activated protein kinases (MAPKs) pathways.

**Results:**

Pretreatment with spermidine prior to LPS treatment significantly inhibited excessive production of NO and PGE_2 _in a dose-dependent manner, and was associated with down-regulation of expression of inducible nitric oxide synthase (iNOS) and cyclooxygenase-2 (COX-2). Spermidine treatment also attenuated the production of pro-inflammatory cytokines, including IL-6 and TNF-α, by suppressing their mRNA expressions. The mechanism underlying spermidine-mediated attenuation of inflammation in BV2 cells appeared to involve the suppression of translocation of NF-κB p65 subunit into the nucleus, and the phosphorylation of Akt and MAPKs.

**Conclusions:**

The results indicate that spermidine appears to inhibit inflammation stimulated by LPS by blocking the NF-κB, PI3K/Akt and MAPKs signaling pathways in microglia.

## Background

Microglia are glial cells that function as the prime effector cells in the immune defense and inflammatory responses in the central nervous system (CNS) [1-3]. These cells are activated in response to environmental stress and produce various bioactive molecules, including nitric oxide (NO), prostaglandin E_2 _(PGE_2_), reactive oxygen species, and pro-inflammatory cytokines, such as interleukin (IL)-1β, IL-6, and tumor necrosis factor (TNF)-α, which function to restore CNS homeostasis by clearing damaged cells and debris [4,5]. However, prolonged microglial activation can cause chronic neuroinflammation and promote neuronal injury due to increased production of neurotoxic pro-inflammatory mediators, and can eventually lead to neuronal death [1,2,6]. This is a common characteristic found in several neurodegenerative diseases [7,8]. Control of microglial activation and subsequent suppression of the production of neurotoxic pro-inflammatory molecules would therefore be an effective therapeutic option for treatment of various neurodegenerative diseases.

Naturally occurring polyamines such as spermidine, spermine, and their precursor putrescine, are thought to play several important control functions in cells, ranging from basic DNA synthesis to regulation of cell proliferation and differentiation [9-11]. Chemically, polyamines are cationic molecules with positive charges that enable electrostatic interactions with polyanionic macromolecules within living cells [12-14]. Several recent studies have suggested that polyamines exert multiple effects including anti-oxidant and anti-inflammatory benefits. For example, Kitagawa and colleagues reported that spermine inhibited the PGE_2 _synthesis and inhibited lipid peroxidation [15-18]. Merentie et al. [19] showed in a pancreatitis model that polyamines could prevent damage to membrane structure caused by activated oxygen radicals by preventing the production of TNF-α and IL-6 production.

Spermidine is a ubiquitous polycation that is synthesized from putrescine and serves as a precursor of spermine. The pancreas is the richest source of spermidine in the body. Eisenberg et al. [20] indicated that an exogenous supply of spermidine prolongs the life span of several model organisms, and significantly reduces age-related oxidative protein damage in mice. These responses suggest that spermidine may act as a universal anti-aging drug. However, the actual molecular mechanisms or signal transduction cascades that underlie spermidine-induced responses, such as anti-inflammatory effects, have not yet been clarified. The present study was designed to evaluate the anti-inflammatory effects of spermidine following lipopolysaccharide (LPS) stimulation of BV2 microglial cells.

## Methods

### Cell culture and cell viability assay

The BV2 immortalized murine microglial cells constructed by infecting primary microglia with a v-raf/v-myc oncogene-carrying retrovirus (J2) were provided by Prof. I.W. Choi (Inje University, Busan, Republic of Korea). The cells were cultured in Dulbecco's modified Eagle's medium (DMEM, Gibco-BRL, Grand Island, NY) supplemented with 10% fetal bovine serum (FBS), penicillin (100 units/ml), and streptomycin (100 μg/ml). Cells were maintained in a humidified incubator with 5% CO_2_. Spermidine was purchased from Sigma-Aldrich Chemical Co. (St. Louis, MO) and dissolved in dimethyl sulfoxide (DMSO, Sigma-Aldrich) as a 1 M stock solution, and dilutions were made in DMEM. The final concentration of DMSO in the medium was less than 0.05% (vol/vol) which showed no influence on cell growth. In all experiments, cells were pretreated with the indicated concentrations of spermidine for 1 h before addition of LPS (0.5 μg/ml, Sigma-Aldrich). The MTT [3-(4,5-dimethylthiazol-2-yl)-2,5-diphenyltetrazolium bromide, Sigma-Aldrich] reduction assay was used for determination of cell viability. In brief, BV2 cells (3 × 10^5 ^cells/well) were seeded and treated with various reagents for the indicated time periods. After various treatments, the medium was removed and the cells were incubated with 0.5 mg/ml of MTT solution. After incubation for 2 h at 37°C and 5% CO_2_, the supernatant was removed and formation of formazan was measured at 540 nm with a microplate reader (Dynatech MR-7000; Dynatech Laboratories, Chantilly, VA).

### Isolation of total RNA and reverse transcription-PCR

Total RNA was prepared using TRIzol reagent (Invitrogen, CA) and primed with random hexamers for synthesis of complementary DNA using M-MLV reverse transcriptase (Promega, Madison, WI), according to the manufacturer's instructions using DNAse I (1 U/μg RNA) pretreated total mRNA. Single stranded cDNA was amplified by polymerase chain reaction (PCR) with primers for inducible nitric oxide synthase (iNOS), cyclooxygenase (COX)-2, IL-1β, TNF-α, and glyceraldhyde-3-phosphate dehydrogenase (GAPDH). The following PCR conditions were applied: GAPDH: 18 cycles of denaturation at 94°C for 30 s, annealing at 57°C for 30 s, and extension at 72°C for 30 s; iNOS, COX-2, IL-6, and TNF-α: 25 cycles of denaturation at 94°C for 30 s, annealing at 52°C for 30 s, and extension at 72°C for 30 s. GAPDH was used as an internal control to evaluate relative expression of COX-2, iNOS, IL-6, and TNF-α

### Western blot analysis

Cells were washed with PBS three times, placed at a temperature of 4°C, and lysed for 30 min in lysis buffer (20 mM sucrose, 1 mM EDTA, 20 μM Tris-Cl, pH 7.2, 1 mM DTT, 10 mM KCl, 1.5 mM MgCl_2 _and 5 μg/ml aprotinin). Lysates were then centrifuged at 12,000 rpm at 4°C. The protein concentration was measured using a Bio-Rad protein assay (Bio-Rad Lab., Hercules, CA) according to the manufacturer's instructions. Equal amounts of protein (30-50 μg) were separated electrophoretically using 8-10% sodium dodecyl sulfate (SDS)-polyacrylamide gel electrophoresis; the gel was then transferred to 0.45 μm polyvinylidene fluoride (PVDF: Millipore, Bedford, MA). Membranes were soaked in blocking buffer (5% skimmed milk) and then incubated with primary antibodies. After thorough washing with PBST, horseradish peroxidase conjugated antibodies were applied and immune complexes were then visualized using the enhanced chemiluminescence (ECL) detection system according to the recommended procedure (Amersham). In a parallel experiment, cells were washed with ice-cold PBS and scraped; and cytoplasmic and nuclear proteins were then extracted using NE-PER^® ^Nuclear and Cytoplasmic Extraction Reagents (Pierce Biotechnology, Rockford, IL). For Western blot analysis, rabbit anti-human iNOS, COX-2, p65, and IκB-α polyclonal antibodies were purchased from Santa Cruz Biotechnology (Santa Cruz, CA). Antibodies against extracellular signal-regulated kinase (ERK), phosphorylated (p)-ERK, p38 mitogen-activated protein kinase (MAPK), p-p38 MAPK, c-Jun N-terminal kinase (JNK), p-JNK, Akt, p-Akt, and lamin B were purchased from Cell Signaling Technology (Danvers, MA). The peroxidase-labeled donkey anti-rabbit immunoglobulin and peroxidase-labeled sheep anti-mouse immunoglobulin were purchased from Amersham Corp. (Arlington Heights, IL).

### Nitrite determination

Levels of NO in culture supernatants were measured by use of the Griess reaction. BV2 cells (4 × 10^5 ^cells/ml) were seeded in six-well plates and stimulated for 24 h with LPS in either the presence or absence of various concentrations of spermidine. Following LPS stimulation, 100 μl of the conditioned culture medium from each sample was mixed with the same volume of Griess reagent [1% sulfanilamide/0.1% N-(1-naphthyl)-ethylenediamine dihydrochloride/2.5% H_3_PO_4_]. NO concentration was determined by measurement of absorbance at 540 nm using a microplate spectrophotometer (Dynatech MR-7000). Nitrite concentration was calculated with reference to a standard curve of sodium nitrite generated by known concentrations [21].

### Measurement of PGE_2_

BV2 cells were plated at a density of 4 × 10^5 ^cells/ml in a six-well cell culture plate and incubated with various concentrations of spermidine in either the presence or absence of LPS (0.5 μg/ml) for 24 h. Following the manufacturer's instructions, a volume of 100 μl of culture-medium supernatant was collected for determination of PGE_2 _concentration by ELISA (Cayman, MI) [22].

### Enzyme-linked immunosorbent assay (ELISA)

Following the manufacturer's instructions, the levels of cytokines, IL-6, and TNF-α, were measured by use of ELISA kits (R&D Systems, Minneapolis, MN). Absorbance was determined at 450 nm using a microplate reader [23].

### Immunofluorescence analysis

For detection of NF-κB p65 translocation, cells were grown on glass coverslips for 24 h and then treated with 0.5 μg/ml LPS, which were either pretreated or not pretreated with spermidine for 60 min. Cells were fixed with 3.7% paraformaldehyde, treated with 0.2% Triton X-100, and blocked with 2% bovine serum albumin (BSA, Sigma-Aldrich). Cells were then sequentially incubated with anti-NF-κB p65 antibody, FITC-conjugated donkey anti-rabbit IgG, and 4,6-diamidino-2-phenylindole (DAPI, Sigma-Aldrich) solution, and examined using a fluorescence microscope (Carl Zeiss, Germany).

### Statistical analysis

Data are presented as the mean ± SD of at least three separate experiments. Comparisons between the two groups were analyzed using the Student's *t*-test. *P *values less than 0.05 were considered statistically significant.

## Results

### Spermidine inhibits NO and PGE_2 _production in LPS-stimulated BV2 microglia

**Figure 1:**
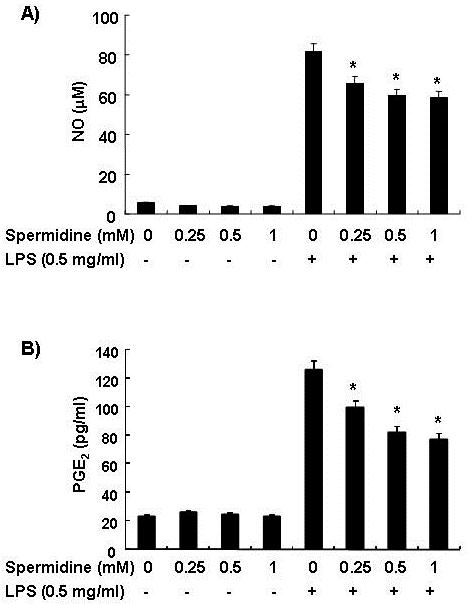
**Effects of spermidine on LPS-induced NO and PGE_2 _production in BV2 microglia**. (A) Cells were treated with the indicated concentrations of spermidine 1 h before a 24 h LPS treatment. Amounts of NO were determined using Griess reagent and a standard curve was created using NaNO_2 _in culture medium. Control values were obtained in the absence of LPS or spermidine. (B) Following the manufacturer's instructions, levels of PGE_2 _in the media were detected using a specific enzyme immunoassay. Each value indicates the mean ± S.D. of three independent experiments. *P < 0.05 indicates a significant difference from cells treated with LPS in the absence of spermidine.

We first examined whether spermidine could regulate production of NO and PGE_2 _produced by microglia. The effect of spermidine on LPS-induced NO and PGE_2 _production was studied by pretreating cells with spermidine for 1 h prior to LPS stimulation for 24 h. The NO and PGE_2 _levels in the cell culture media were then measured. As shown in Figure 1, LPS alone markedly induced NO and PGE_2 _production, compared to the control; however, both NO and PGE_2 _production by LPS-activated cells were significantly inhibited by spermidine in a concentration-dependent manner. Pretreatment with spermidine therefore significantly suppressed expression of LPS-mediated pro-inflammatory mediators. The selected concentrations of spermidine used in our experiment did not exhibit any significant cytotoxicity even at the highest concentration (1 mM) for up to 24 h of incubation, in all cases, cell viability remained above 95% as determined by the MTT assay (data not shown). This confirmed that the observed inhibition of NO and PGE_2 _production in LPS-stimulated BV2 cells was not due to a cytotoxic action of spermidine or LPS.

### Spermidine attenuates expression of LPS-stimulated iNOS and COX-2 mRNA and protein

**Figure 2:**
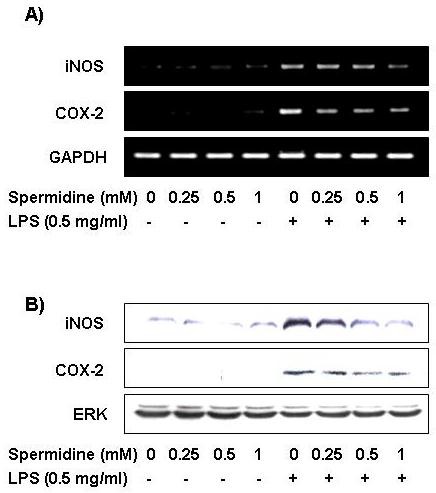
**Effects of spermidine on LPS-induced expression of iNOS and COX-2 mRNA and protein in BV2 microglia**. (A) BV2 cells were pretreated with different concentrations of spermidine for 1 h followed by LPS stimulation for another 6 h. Total RNAs were isolated, and mRNA levels of iNOS and COX-2 were measured by RT-PCR. GAPDH expression was used as an internal control. (B) After 24 h treatment, the cells were lysed and the cellular proteins (50 μg) were separated by electrophoresis on SDS-polyacrylamide gels and transferred onto nitrocellulose membranes. The membranes were probed with the indicated antibodies and the proteins were visualized using an ECL detection system. ERK was used as an internal control.

We carried out RT-PCR and Western blot analyses to investigate the question of whether inhibition of NO and PGE_2 _production were associated with decreased levels of iNOS and COX-2 expression. As shown in Figure 2A and 2B, iNOS and COX-2 mRNA was detected 6 h after LPS treatment, and the enzyme proteins were detected in whole cell lysates 24 h after LPS treatment. However, spermidine treatment of LPS-stimulated BV2 microglia significantly decreased both iNOS and COX-2 mRNA and protein levels. Spermidine-induced reduction in expression of iNOS and COX-2 was apparently responsible for the observed inhibition of NO and PGE_2 _production.

### Spermidine suppresses production of inflammatory cytokines in LPS-stimulated BV2 microglia

**Figure 3:**
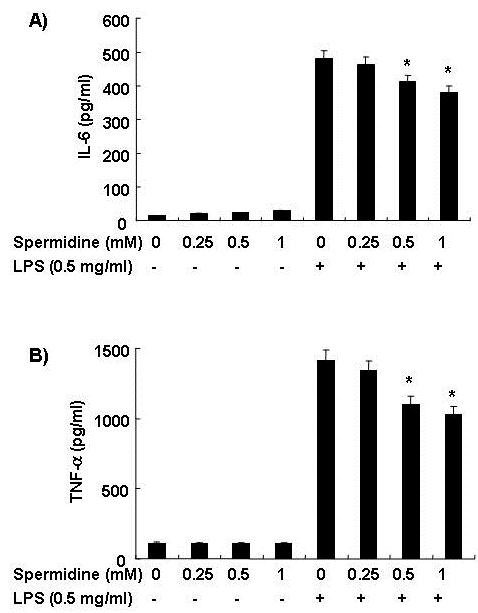
**Effects of spermidine on pro-inflammatory cytokine production in LPS-stimulated BV2 microglia**. Cells were pretreated with the indicated concentrations of spermidine for 1 h before LPS treatment. Following incubation for 24 h, the supernatants were analyzed for IL-6 (A) and TNF-α (B) content. Each value indicates the mean ± S.D. of three independent experiments. *P < 0.05 indicates a significant difference from cells treated with LPS in the absence of spermidine.

**Figure 4:**
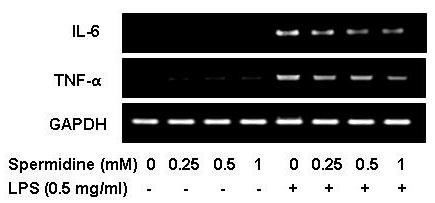
**Effects of spermidine on expression of IL-6 and TNF-α mRNA in LPS-stimulated BV2 microglia**. Cells were pretreated with the indicated concentrations of spermidine for 1 h before LPS treatment, and the total RNAs were isolated at 6 h after LPS treatment. The levels of IL-6 and TNF-α mRNA were determined by RT-PCR. GAPDH was used as internal control.

We next investigated whether spermidine suppresses production of pro-inflammatory cytokines, such as IL-6 and TNF-α, in LPS-stimulated BV2 cells. For this study, BV2 microglia were incubated with spermidine in the absence or presence of LPS for 24 h, and the cytokine levels in the culture supernatants were evaluated. As indicated in Figure 3A and 2B, the production of IL-6 and TNF-α induced by LPS treatment was significantly decreased by treatment with spermidine. In a parallel experiment, using RT-PCR, we studied the effects of spermidine on LPS-induced IL-6 and TNF-α mRNA expression. As shown in Figure 4, the levels of expression of IL-6 and TNF-α mRNA also decreased in response to spermidine treatment. These results suggest that spermidine is effective in suppression of pro-inflammatory cytokine production in activated microglia through alteration of the transcription levels of IL-6 and TNF-α.

### Spermidine blocks NF-κB activity in LPS-stimulated BV2 microglia

**Figure 5:**
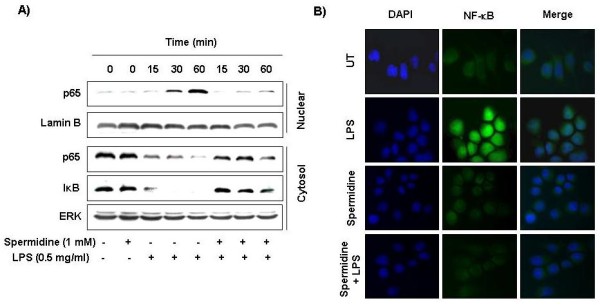
**Effects of spermidine on NF-κB activity in LPS-stimulated BV2 microglia**. (A) Cells were pretreated with the indicated concentrations of spermidine 1 h before LPS treatment for the indicated times. Total cytosolic (30 μg) or nuclear (30 μg) proteins were separated on 10% SDS-polyacrylamide gels, followed by Western blotting using anti-NF-κB p65 and IκB-α. Proteins were visualized using an ECL detection system. ERK and lamin B were used as internal controls. (B) NF-κB p65 was localized by fluorescence microscopy after immunofluorescence staining with NF-κB p65 antibody (green). Cells were stained with DAPI for visualization of nuclei (blue). Results are representative of those obtained from three independent experiments.

Activation of NF-κB is the key event for the induction of all major pro-inflammatory mediators. Therefore, we used Western blotting and immunofluorescence microscopy to examine the effect of spermidine on NF-κB activation. As shown in Figure 5A, immunoblotting results indicated that stimulation of cells with LPS induced the degradation of IκBα, and translocation of the NF-κB p65 subunit from the cytosol to the nucleus. However, the LPS-induced IκB degradation was inhibited following a 30 min of exposure to spermidine. Spermidine treatment also inhibited nuclear translocation of the NF-κB p65 protein. Immunofluorescence microscopy results indicated similar responses (Figure 5B). These results suggest that spermidine may inhibit NF-κB activation in BV2 microglia cells by suppression of IκB degradation and nuclear translocation of NF-κB.

### Spermidine reduces LPS-induced phosphorylation of Akt and MAPKs in LPS-stimulated BV2 microglia

**Figure 6:**
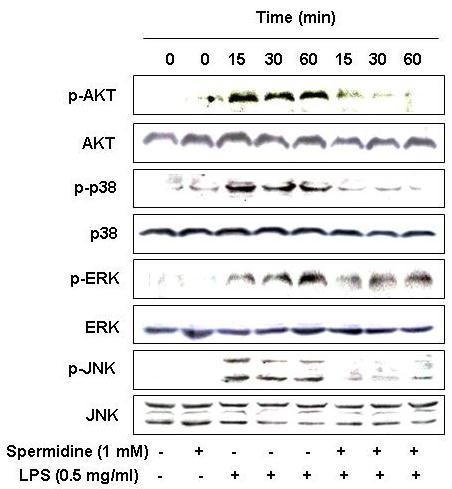
**Effects of spermidine on Akt and MAPKs activation induced by LPS in BV2 microglia**. Cells were treated with different concentrations of spermidine 1 h before LPS treatment for the indicated times. Total proteins (50 μg) were separated on 10% SDS-polyacrylamide gels, followed by Western blotting using the indicated antibodies. Results are representative of those obtained from three independent experiments.

We investigated an alternative intracellular mechanism potentially responsible for the inhibitory effect of spermidine on inflammatory mediators by examining the effect of spermidine on Akt and MAPKs signaling pathways. As shown in Figure 6, phosphorylation of Akt was increased within 15 min after LPS stimulation and spermidine pretreatment resulted in marked blockage of this phosphorylation. Stimulation of BV2 cells with LPS led to rapid activation of p38 MAPK, ERK, and JNK, with peak levels of each phospho-MAPK occurring 15 to 60 min after addition of LPS. Spermidine pretreatment significantly inhibited this phosphorylation of MAPKs in LPS-stimulated BV2 microglia (Figure 6). This finding suggests that spermidine is capable of disrupting key signal transduction pathways such as Akt and MAPKs that are activated by LPS in BV2 microglia. This then prevents production of pro-inflammatory mediators.

## Discussion

Inflammation plays an important role in the pathology of neurodegenerative disorders in the brain. In particular, neuroinflammation with prolonged activation of microglial cells leads to an increased production of pro-inflammatory mediators and cytokines. This contributes to neuronal dysfunction and neuronal loss and ultimately leads to neuronal cell death [1,2,6]. Therefore, inhibitors of these inflammatory molecules have been considered as candidate anti-inflammatory drugs for alleviation of the progression of neurodegenerative disease caused by activation of microglia [8,24,25]. In the present study, we demonstrated that spermidine treatment of activated BV2 microglial cells resulted in significant inhibition of the production of the LPS-induced pro-inflammatory mediators (NO and PGE_2_) and cytokines, (including TNF-α and IL-6). These effects were accompanied by down regulation of NF-κB, and inactivation of PI3K/Akt and MAPKs signaling pathways. Therefore, inhibition of pro-inflammatory molecules by spermidine, as shown in the present study, could be beneficial in the treatment of neurodegenerative diseases.

NO is an important regulatory mediator that involved in cell survival and death, and it also exerts a number of pro-inflammatory effects in several physiological and pathological processes. High levels of NO are produced from L-arginine by iNOS in the brain by prolonged activation of microglial cells, and this response is associated with the progression of various neurodegenerative diseases [26]. Similarly, another well-known inflammatory mediator, PGE_2_, which is generated from arachidonic acid via the action of COXs, contributes to the development of many chronic inflammatory diseases [27,28]. Overproduction of PGE_2 _in response to various inflammatory stimuli is associated with up-regulation of COX-2 and progression of inflammation. Overall, COX-2 has emerged as one of the major players in brain inflammation, and increased COX-2 expression is believed to contribute to neurodegeneration [29,30]. Therefore, any substance that can attenuate expression of iNOS and COX-2 could be beneficial for preventing and delaying the progression of neurodegenerative disease.

In the present study, we found that spermidine treatment of LPS-stimulated BV2 cells effectively decreased iNOS and COX-2 mRNA and protein expression and the release of their respective end-products, NO and PGE_2 _(Figure 1 and 2). These effects were not due to any cytotoxicity of spermidine, as verified by the MTT assay. Thus, the observed inhibitions of NO and PGE_2 _production may be attributed to the suppression of the transcription of iNOS and COX-2 mRNA and subsequent reduction in protein expressions. Therefore, spermidine may impart beneficial effects by attenuation of microglial activation and subsequent production of inflammatory neurotoxins.

Pro-inflammatory cytokines, such as IL-1β, IL-6 and TNF-α, are the initiators of the inflammatory response and the mediators of the development of chronic inflammatory diseases. Therefore, overproduction of pro-inflammatory cytokines can be considered as a histopathological hallmark of various neurological diseases in the brain [5,17,31]. The present demonstrated that spermidine significantly inhibited the generation of IL-6 and TNF-α in LPS-stimulated BV2 microglia in a concentration-dependent manner by suppressing mRNA expressions (Figure 3 and 4). This indicated that the inhibitory action of spermidine on production of inflammatory mediators occurs at the transcriptional level.

The transcription factor NF-κB is known to play a critical role in controlling most inflammatory responses due to its ability to induce transcription of pro-inflammatory genes such as inducible enzymes, iNOS and COX-2 and pro-inflammatory cytokines including TNF-α and IL-6 [32-34]. The activity of NF-κB is suppressed in the cytoplasm in either the homodimer or a heterodimer form, while it is complexed with an inhibitory IκB protein. The activation of NF-κB results in the phosphorylation, ubiquitination, and proteasome-mediated degradation of the IκB proteins, followed by nuclear translocation [35,36]. The NF-κB dimers are then free to translocate to the nucleus and activate target genes. Recently, involvement of the phosphoinositide 3-kinase (PI3K)/Akt pathway has also been demonstrated in the expression of inflammatory mediators in microglia through activation of NF-κB by IκB degradation [37]. Therefore, blocking the NF-κB transcriptional activity and Akt activation has been identified as an important target for the treatment of inflammatory diseases. In the present study, we found that spermidine treatment attenuated the phosphorylation and degradation of IκB in cytosol. The translocation of NF-κB factor p65, which is normally translocated from the cytoplasm to the nucleus after exposure to LPS, was also strongly inhibited by spermidine (Figure 5). The increase in phosphorylation of Akt normally seen after exposure to LPS was also markedly inhibited by spermidine (Figure 6). These results suggest that the effects of spermidine on the production of inflammatory mediators and cytokines are at least partially mediated by the suppression of the NF-κB and PI3K/Akt signaling pathway.

In addition to NF-κB, LPS is a potent activator of the MAPKs pathway, which is the other major extracellular signal transduction pathway stimulated by inflammatory mediators. Once activated, MAPKs modulate the functional responses of cells through phosphorylation of transcription factors and activation of other kinases [38,39]. The MAPKs are also known to be involved in LPS-induced production of COX-2 and iNOS *via *control of NF-κB activation in microglial cells [40,41], which indicates that MAPKs function as important targets for anti-inflammatory molecules. When investigated the effects of spermidine on the LPS-induced phosphorylation of MAPKs, we found that spermidine treatment significantly inhibited LPS-stimulated phosphorylation of p38 MAPK, ERK and JNK (Figure 6). This suggested that the observed anti-inflammatory effects were also due to inhibition of the MAPKs signaling pathway.

## Conclusion

In conclusion, the present study has revealed that spermidine treatment of BV2 microglial cells inhibited LPS-induced NO and PGE_2 _production by suppressing iNOS and COX-2 mRNA and protein expression. Spermidine also inhibited the production of pro-inflammatory cytokines, such as TNF-α and IL-6, by suppressing their transcriptional activity. These effects were exerted by attenuation of translocation of NF-κB from the cytoplasm to the nucleus, which was accompanied by blocking of PI3K/Akt and MAPKs pathways. This suggests that spermidine may have substantial therapeutic potential for treatment of neurodegenerative diseases that are accompanied by microglial activation.

## Competing interests

The authors declare that they have no competing interests.

## Authors' contributions

HYP performed research and wrote the manuscript. YHC contributed to the experimental design, data interpretation, editing, and submission of this manuscript. All authors read and approved the final manuscript.
